# Novel insights on multilevel factors that affect the dynamic course of financial toxicity in cancer patients

**DOI:** 10.1093/jncics/pkae020

**Published:** 2024-04-09

**Authors:** Grace L Smith

**Affiliations:** Department of GI Radiation Oncology, Department of Health Services Research, University of Texas, MD Anderson Cancer Center, Houston, TX, USA

Financial toxicity is one of the most ubiquitous impacts of disease and treatment in individuals who have had a cancer diagnosis. The financial impact stems from the costly effects of treatment, including direct out-of-pocket spending, time, and toxicities associated with cancer treatment, along with effects of disease—long-term functional or cognitive impairments and disability that can impact employment as well as direct medical costs related to cancer surveillance. These financial impacts can extend through all phases of cancer care and survivorship. A recent systematic review reported that the pooled incidence of financial toxicity, defined broadly, in individuals with breast cancer was 35.3% (95% confidence interval [CI] = 27.3% to 44.4%) for high-income countries like the United States (1), an estimate corroborated by the analysis by Berlin et al. (2) in this issue of the Journal, which focuses on select aspects of financial toxicity, including catastrophic health expenses and nonemployment in a sample of breast cancer patients and survivors derived from the Medical Expenditure Panel Survey.

Findings from this study provide novel insights that highlight multilevel, dynamic aspects of financial toxicity that impact individuals with a cancer diagnosis more frequently than individuals without a history of cancer. Shifting temporal patterns elucidated by Berlin et al. (2) demonstrate that secular trends influence or even modify the way financial toxicity materializes for populations and subgroups of cancer patients. In the current study, despite evidence that the Affordable Care Act (ACA) successfully enhanced health insurance accessibility over time for an increased proportion of the population with breast cancer, during the same period, the proportion facing catastrophic health expenditures statistically significantly rose. Notably, prior to ACA (2005-2009), one-quarter of the study population with breast cancer experienced health expenditures exceeding 10% of their annual income, and this effect steadily rose to more than one-third of the population with breast cancer experiencing catastrophic health expenditures in the post-ACA era (2013-2019). Underlying secular trends that could contribute to the temporal pattern of catastrophic health expenditures’ impact delineated by Berlin et al. (2) include the overall rise in prices of anticancer therapies as well as in an increasing reliance on cost sharing during the study period, especially for younger patients who are ineligible for Medicare. Together, these forces have placed escalating out-of-pocket cost burdens on patients (3-5). Anticipated closure of Medicare coverage gaps for costs of prescription medications by policies such as the 2022 Inflation Reduction Act has been anticipated to have a potential to mitigate the medication cost–related personal burden of financial toxicity, at least among Medicare-eligible cancer populations. However, these policy shifts are more expected to benefit older populations. Whether policy shifts may also be able to render a favorable impact on the overall proportion and severity of younger populations experiencing cancer-related financial toxicity will need ongoing examination. Besides policy impacts, the current impact on cancer-related financial toxicity of other societal factors, such as political, social, and economic trends in the post–COVID-19 pandemic era, is yet to manifest fully (6). Recently published data by Thom et al. (6) on the basis of an online sample of young adult cancer survivors, however, provide initial data suggestive that exposure to negative economic events in the peripandemic period, and especially exposure to multiple contemporary risk factors, including work disruptions and decreases in job security or pay, job loss, and increases in credit card debt, renders younger cancer survivors at particular risk for cancer-related financial toxicity.

The study by Berlin et al. (2) further underscores how dynamic individual factors impacting financial toxicity trajectory are superimposed on dynamic societal influences. This superimposition of different levels of modifiers adds complexity to a comprehensive understanding of the problem of financial toxicity. The Medical Expenditure Panel Survey dataset did not identify clinical and treatment details of the study sample, including time since diagnosis, stage of diagnosis, treatment course, and completion of vs ongoing breast cancer–related care. However, given that the sample was population based, it is critical to recognize that this study provides a broad snapshot of how financial toxicity appears to impact individuals over the entire spectrum across phases of care from cancer diagnosis to long-term survivorship. For example, individuals with breast cancer were more likely to have experienced missed workdays and any illness-related nonemployment as well as full-year nonemployment than those without cancer—reflecting the relevance short-term and more enduring aspects of financial toxicity during an individual’s initial experience with disease and treatment and ultimately over survivorship. Less is known about longitudinal patterns of cancer-related financial toxicity. Notably, a recent analysis by Kircher et al. (7) demonstrated in a prospective cohort of individuals with colorectal cancer a statistically significant temporal trajectory of financial toxicity over the first 12 months from cancer diagnosis, with financial toxicity generally improving over this period, though heterogeneously across different patient subgroups. For example, employment status, education level, race, and primary insurance type were patient sociodemographic characteristics that appeared to modify the longitudinal financial toxicity trajectory, with adversity (eg, unemployment, lower education) impeding recovery from financial toxicity. The individual psychological characteristic of degree of self-efficacy also appeared to be a modifier of financial toxicity longitudinal patterns, with patients who reported better self-efficacy for managing the physical, emotional, and practical effects of chronic disease more likely to improve in financial toxicity severity over time. Beyond the initial 12 months from cancer diagnosis, and in other disease sites, data in the scientific literature remain lacking. Thus, the gaps in the longitudinal evidence on financial toxicity’s dynamic trajectory certainly warrant future investigation.

With the emerging understanding of the modifying effects of societal and individual factors on financial toxicity, several additional repercussions, scientifically and clinically, emerge. Financial toxicity is already understood to be a complex multidimensional construct, with direct material, coping or behavioral, and psychological domains. Scientifically, several instruments have been derived to measure or screen for financial toxicity, including the Comprehensive Score for financial Toxicity, Economic Strain and Resilience in Cancer, and European Organisation for Research and Treatment of Cancer Quality of Life questionnaire C30 financial difficulties measure, all demonstrating strong validity, reliability, and prognostication for economic and care delivery outcomes in individuals with cancer in studies over the last decade. Scientifically, it remains imperative for the academic research community to remain committed to continuous iteration in the accurate measurement of financial toxicity, potentially varying or modifying these instruments in coming decades as the landscape of macro-environmental factors potentially shifts meaningfully. An exemplar is the recent development of tools to measure patient-reported symptom severity specific to immunotherapy (including the MD Anderson Symptom Inventory–Immunotherapy module, as one example among several such tools). The advent of these measures recognizes the groundbreaking impact and vast uptake of immunotherapy as truly transformative of not only cancer therapy but also the relevant profile of acute and chronic physical symptoms and related quality-of-life profile that cancer patients who receive immunotherapy experience.

Finally, there exists a key clinical implication of the dynamic nature of financial toxicity. For oncology provider teams integrating the financial aspects with a patient’s cancer treatment plan or directly intervening on financial toxicity during cancer care, repeated evaluation of financial toxicity with an awareness that the severity along with the aspects of this toxicity could morph for an individual patient over time (8). Establishing a practical, systematic approach in cancer care delivery for iterative evaluation of and intervention on the different early and late aspects of financial toxicity as well as downstream impacts is needed in practice. Overly simplistic characterization of financial toxicity as uniform or static is likely insufficient to optimize practical intervention for cancer patients and survivors in clinical settings. Therefore, this important analysis by Berlin et al. (2), though spanning data collected on patients between 2005 and 2019, elucidates a deeper understanding of the phenomenon of financial toxicity still directly relevant to present and impending experiences of patients and survivors with cancer.

## Data Availability

No new data were generated or analyzed for this editorial.
